# Development and evaluation of an early detection intervention for mouth cancer using a mass media approach

**DOI:** 10.1038/sj.bjc.6605395

**Published:** 2009-12-03

**Authors:** D Eadie, A M MacKintosh, S MacAskill, A Brown

**Affiliations:** 1 Centre for Tobacco Control Research, University of Stirling and The Open University, Stirling FK9 4LA, UK

**Keywords:** mouth cancer, early detection, mass media, evaluation

## Abstract

**Background:**

Scotland has a high incidence of mouth cancer, but public awareness and knowledge are low compared with other cancers. The West of Scotland Cancer Awareness Project sought to increase public awareness and knowledge of mouth cancer and to encourage early detection of symptoms among an at-risk population of people aged over 40 years from lower socio-economic groups using a mass media approach. The media campaign aimed to increase people's feelings of personal risk, while also enhancing feelings of efficacy and control. To achieve this, a testimonial approach (using real people to tell their own stories) was adopted.

**Methods:**

Campaign impact and reach was assessed using in-home interviews with a representative sample of the target population in both the campaign area and controls outside of the target area. Surveys were conducted at three stages: at baseline before the campaign was launched, and at 7 and 12 months thereafter.

**Results:**

Awareness of media coverage was higher at both follow-up points in the intervention area than in the control area, the differences largely being accounted for by television advertising. The campaign had a short-term, but not a long-term impact on awareness of the disease and intention to respond to the symptoms targeted by the campaign. Awareness of two of the symptoms featured in the campaign (ulcers and lumps) increased, post-campaign, among the intervention group.

**Conclusions:**

While the study provides evidence for the effectiveness of the self-referral model, further work is needed to assess its ability to build public capacity to respond appropriately to symptoms and to compare the cost-effectiveness of a mass media approach against alternative communication approaches and more conventional mass screening.

Mouth cancer is defined as cancers of the lip, tongue, oral cavity, oropharynx, hypopharynx and piriform sinus. Globally, mouth cancer is the 12th most common malignant tumour. In the United Kingdom, ∼5300 people are diagnosed with mouth cancer each year and about half of those will die from the disease. Scotland has a higher incidence of mouth cancer than any other country in the United Kingdom: over 600 new cases are diagnosed each year (Office for National Statistics, 2009). Epidemiological data suggests a clear link between smoking, heavy drinking and deprivation and the onset of mouth cancer. In addition, mouth cancer is most common in older people, with 86% of cases occurring in people over the age of 50 years (Office for National Statistics, 2009).

Research suggests that survival rates could be improved by up to 30%, and quality of life enhanced, if people present with early symptoms of the disease (Schrijvers *et al*, 1995). Many pre-cancerous malignancies can be detected by physical inspection of the mouth. However, nearly two in five (38.7%) of adults in Scotland are not registered with a dentist, with registration rates declining sharply above the age of 55 years (ISD, 2009). Furthermore, public awareness of the disease and its symptoms is poor. An unpublished survey conducted by Market Research UK in 2001 revealed that only 6% of adults in the west of Scotland were aware of mouth cancer (unprompted) and 54% were unable to name a single symptom. These findings are consistent with those found in other population studies (Cruz *et al*, 2002; Hay *et al*, 2002; Horowitz *et al*, 2002; West *et al*, 2006).

It is speculated that infrequent coverage of mouth cancer in the popular media may help to explain the public's lack of knowledge about the disease. Although cancer receives a large amount of media attention, coverage tends to emphasise cancer deaths and treatment in preference to prevention and early detection (Signorielli, 1990), and it also gives certain cancers, most notably breast cancer, greater coverage (Marino and Gerlach, 1999). In contrast, low media coverage of mouth cancer helps to explain poor public awareness and understanding of the disease in the United States. A 10-year study of newspaper and magazine coverage of mouth cancer in the United States found only 50 articles and news items on mouth cancer (Canto *et al*, 1998). While the majority of these articles mentioned at least one-risk factor, only half made any reference to symptoms of the disease and substantially fewer suggested visiting a health professional for advice.

The strong evidence base for the ability of media campaigns to promote improvements in health awareness and knowledge (Campion *et al*, 1994; Hafstad *et al*, 1997) and to increase participation rates in mass screening programmes (Gregorio *et al*, 1990; Shelley *et al*, 1991) has focussed attention on the potential to combine these strengths in campaigns promoting the early detection of disease. While mass screening programmes are aimed at all members of the at-risk population, early detection aims to encourage only those who experience relevant disease symptoms to self-present to an appropriate health professional for advice and possible onwards referral to specialist diagnostic and treatment services. However, unlike mass screening, the effectiveness of early detection depends on raising public awareness of the symptoms and equipping people with the ability to identify the symptoms and respond appropriately.

This paper reports on the development and evaluation of a mass media intervention to increase awareness, knowledge and early detection for mouth cancer in the west of Scotland. The West of Scotland Cancer Awareness Project (WoSCAP) was targeted at an at-risk population of people aged 40 years and over from lower socio-economic groups across the west of Scotland. The primary aim of the campaign was to encourage people to present to a local general dental practitioner if they experienced symptoms that might indicate the early onset of mouth cancer. The campaign's impact on patient presentation to primary healthcare teams will be reported separately. The campaign, the first of its kind in the United Kingdom, brought together five local regional health boards with a combined population of over 2.5 million, nearly half of Scotland's entire population. The partnership between the health boards offered a number of structural and economic benefits, most notably increased media buying power, access to regional broadcast networks and continuity with centralised treatment services.

A key challenge facing the media campaign was the need to increase public awareness of personal risk and perceived susceptibility, while at the same time avoiding raising unnecessary anxiety and encouraging inappropriate self-referrals from the so-called ‘worried well’. Given the experimental nature of the campaign, there was a deeply felt concern among service planners and providers that a failure to meet demand for diagnostic services could lead to unacceptable delays for those requiring treatment. Consequently, the campaign was grounded in social marketing to ensure campaign development was consumer led and founded on sound communication principles. This involved precise targeting of the at-risk population using formative research and pre-testing techniques to guide the campaign message, tone, imagery, language, etc. (Eadie and Smith, 1995). As an additional safeguard, local monitoring procedures were set in place to ensure that the media schedule could be modified at short notice to moderate demand for diagnostic services.

Careful consideration was also given to the campaign media strategy and channel selection in terms of reach, message delivery and integration with other programme components. There is substantial evidence that people in low-income groups and with lower educational attainment display above average levels of television viewing and radio listening (Social Trends, 1995; Anderson *et al*, 1989). Other research has shown print media to be effective at communicating detailed factual information about cancer (Humphris *et al*, 2001), and direct mail campaigns to be a particularly cost-effective method for encouraging low-income groups to attend for cancer screening (Sugg-Skinner *et al*, 1994). There is also substantial evidence that awareness campaigns, which adopt a multi-faceted approach, for example, combining paid and unpaid media and interpersonal components, reinforce and amplify one another and combine to produce a greater and longer-lasting effect (Fortmann *et al*, 1995; Pentz *et al*, 1997). This evidence base led to an integrated media strategy combining two 40-second advertisements, one on television and the other on local radio, supported by free wall posters, leaflets and direct mail leaflet drops in key target communities. In addition, the project also worked closely with the media to generate news coverage in local and national press and by broadcast media. The total cost of the campaign, including public relations activity, was £264 000.

## Campaign theory

The WoSCAP campaign goals and message strategy were established using social cognitive theory (Bandura, 1977, 1986), which defines human behaviour as being reciprocally determined by personal factors (such as knowledge, skills, self-efficacy, outcome expectations and personal goals) and environmental factors (such as social and institutional norms, and physical environment). A review of the existing evidence base and exploratory research conducted with the target group identified three personal factors responsible for discouraging patients to come forwards for help and advice: poor knowledge, low perceived risk and fear of negative health outcomes, such as being diagnosed with the disease and concerns over effectiveness of treatment.

These factors were critical to setting the campaign goals and the means by which each might be achieved: to raise awareness of the disease and methods of early detection and to trigger help-seeking behaviour. The first goal was an important precursor to action and played to one of the main strengths of media-based communication – the ability to increase audience awareness and knowledge. However, the second goal, developing a persuasive message strategy, presented a particular challenge – to increase people's feelings of personal risk while, at the same time, enhancing feelings of efficacy and control. A means had to be found of bringing these two motivational themes together in a way that was both credible and persuasive. The existing evidence highlights the risks to using overt fear appeals and their ability to evoke maladaptive responses such as avoidance and distortion (Tanner *et al*, 1991; Schoenbachler and Whittler, 1996; Witte *et al*, 1998). Given the fatalism and very real sensitivities that characterised people's feelings about personal intervention and secondary forms of cancer prevention, there was a real risk that using overt fear appeals would undermine any attempts to boost people's confidence to respond proportionately. Instead, the campaign turned to social cognitive theory for a possible solution.

Social cognitive theory proposes that much behaviour is learned from observing the actions of others and the consequences for them, and suggests that the acquisition of new patterns of behaviour can be accelerated by ‘modelling’ the desired behaviour, that is using real people to either explain or show through action how they made the behaviour change (Bandura, 1977, 1986). Sutton *et al* (1995) suggest that social modelling helps the individual master the behaviour and provides them with the confidence to make the change. Formative research carried out with members of the target audience found support for a testimonial approach, using real people to describe in their own terms their symptoms, their responses and the consequences of their actions. The testimonial approach offers a number of benefits. For example, using ordinary people who tell their own stories allows the target audience to identify more directly with the problem, and to reassess the significance of what could be dismissed as relatively innocuous everyday ailments. In this way, the approach provides normative support for the idea of looking out for the early symptoms of the disease. In addition, by selecting personal testimonies, which not only illustrate the positive health outcomes of responding promptly to the symptoms, but also the negative outcomes of failing to respond, the approach provides a credible means of combining the potentially discordant motivational themes of self-efficacy and personal risk.

In addition to influencing personal factors, the WoSCAP campaign also sought to influence environmental factors by using paid advertising to leverage news coverage and local briefing events to encourage primary healthcare professionals to carry the campaign message to key patient populations. In this way, the campaign sought to influence *media* and *institutional* environments to build word-of-mouth communication and to raise the level and standard of public debate around the issue.

## Materials and methods

A three-stage cross-sectional tracking survey with control was conducted to monitor campaign reach (e.g. awareness and response to individual campaign elements) and communication effect (e.g. impact of the campaign on knowledge of early symptoms and propensity to self-refer). A baseline survey was conducted before the launch of the media campaign, with a first follow-up survey 7 months later immediately after the main burst of media activity and a second follow-up completed a further 12 months after that.

Intervention and control samples were selected using a random location quota methodology to be broadly representative of the campaign's target audience: adults aged 40–70 years from social classes C1, C2, D and E. Respondents were recruited on the basis of meeting the socio-demographic requirements and living in postcode sectors with high social disadvantage ratings. A sample of 80 postcode sectors was drawn from non-rural localities within eight health board areas, 50 postcode sectors from the five health boards participating in the campaign and the remaining 30 postcode sectors from three health boards that made up the control area. Within each of the intervention and control areas, postcode sectors were randomly selected with probability proportionate to size and were stratified by health board and disadvantage score. Both samples were drawn from private households within the selected postcode sectors.

Both surveys were administered using face-to-face in-home interviewing techniques and were conducted by a team of professional market research interviewers. Interviewers were assigned a quota of 12 interviews and instructed to obtain a minimum of 10 interviews in each of the 80 allocated areas (target sample 800 interviews). The quota was interlocked on age, sex and social class. A total of 922, 934 and 944 completed interviews were achieved at baseline and first and second follow-up, respectively (Table 1).

The target sample within each of the quota categories was achieved; additional interviews tended to be with female and older respondents and were retained within the survey. Although the sample profile has a higher proportion of females and older respondents than intended, the profiles of the intervention and control samples did not differ.

The exclusion of rural areas and the reliance on non-probability sampling methods means that this sample cannot be said to be completely representative of, or absolutely predictive for, the target population. The sample is, however, large enough to be strongly indicative of the complete target population. It is, therefore, useful to examine the pattern of the results and conduct statistical tests to gain an understanding of the sample as if it were generated by a random selection process.

Within each survey wave, logistic regression has been used to examine differences by intervention status, while controlling for age, gender, social grade, marital status, education level and experience of cancer. To enable interpretation of post-intervention results, where differences already existed at baseline, logistic regression was run with all three survey waves and incorporated a survey stage by intervention status interaction term. A significant interaction term provided an indication that intervention and control samples displayed different patterns across survey waves. If the interaction term was not significant, this indicated that, after controlling for baseline differences, there was no impact from the intervention.

## Results

The results are organised by media coverage of mouth cancer, awareness and perceived incidence of the cancer, awareness of mouth cancer symptoms, salience of mouth cancer symptoms and intention to respond to symptoms of mouth cancer.

### Media coverage

To provide a context for understanding the campaign's impact on awareness and knowledge of the disease, respondents who had heard of mouth cancer were asked whether or not they had come across media coverage about it from a range of sources (see Table 2). Awareness of any source of media coverage was higher, at baseline and both follow-up surveys, among the intervention sample compared with the control sample (27 *vs* 20% (*P*<0.01), 80 *vs* 26% (*P*<0.001) and 40 *vs* 14% (*P*<0.001), respectively). After controlling for the difference at baseline, the post-campaign differences between intervention and control samples remained significant. Television advertising largely accounted for the media coverage (74 *vs* 11% (*P*<0.001) and 30 *vs* 3% (*P*<0.001)) at first and second follow-up, respectively. At the first and second follow-up, fewer than 10% of respondents in the intervention area cited each of the other media, newspapers, radio, poster or booklets/leaflets, as sources of media coverage.

The results also revealed differences in media coverage over time with awareness of any media source increasing between baseline and first follow-up, rising from 27% to 80% in the intervention area and then falling back to 40% by second follow-up. These differences were again attributed in large part to television advertising (the campaign's primary medium), which peaked at 74% at first follow-up and then fell to 30% by second follow-up. These figures provide a reliable measure of campaign recall as no other mouth cancer prevention campaigns using television advertising were running at the time of exposure.

Among the intervention sample prompted, awareness of the TV advertisement was 83% at the first follow-up and 69% at the second follow-up. A substantial proportion of the control sample also indicated awareness of the TV advertisement with 23% aware at the first follow-up and 25% aware at the second follow-up.

### Awareness and perceived incidence of mouth cancer

Respondents were asked to name cancers that they had heard of. They were then given a list of up to 11 cancers and, for each one that they had not already mentioned, were asked whether or not they had heard of it. Table 3 shows awareness of each. Awareness of most cancers was high, 80% or more and there were no differences by intervention status at baseline. At the first follow-up, mouth cancer was the only one which differed by intervention status, with intervention respondents having higher awareness (92 *vs* 85%; *P*<0.01). However, this difference was not maintained at the second follow-up, suggesting that the campaign may have had a short-term effect on awareness of the disease.

A measure of the perceived incidence was also taken for 9 of these 11 cancers (Table 4), by asking respondents how rare or common they thought each cancer was in Scotland. Answers were given on a five-point scale ranging from 1 (very rare) to 5 (very common). Breast and lung cancer had the highest perceived incidence with over 80% of respondents considering these cancers to be very or quite common. Mouth cancer (23% intervention *vs* 12% control (*P*<0.01) at baseline) along with bladder cancer (26% intervention *vs* 19% control (*P*<0.05)) had low perceived incidence relative to other cancers. Throughout all survey stages, intervention respondents were more likely than those from the control sample, to perceive mouth cancer to be very or quite common (*P*<0.01). However, the pattern across time did not differ between intervention and control respondents, and, therefore, provided no indication that the campaign resulted in higher perceived incidence of mouth cancer.

An analysis of these same data by campaign awareness reveals differences for mouth cancer at first follow-up, with those aware of the campaign more likely than those unaware of the campaign, to consider mouth cancer to be very or quite common (26% aware of TV ad *vs* 15% not aware; *P*<0.01). By the second follow-up, perceived incidence did not differ by awareness of the TV advertisement.

### Awareness of symptoms

Understanding of mouth cancer and its detection was measured using an open-ended question, which asked respondents to describe in their own terms the symptoms of the disease. Table 5 describes the data to emerge from an analysis by intervention and control samples, differentiating between those symptoms highlighted by the campaign and those not highlighted.

For many of the symptoms highlighted by the campaign, intervention respondents at baseline were significantly more likely than their control respondents to mention these symptoms (sores, red or white patch, changes that persist and changes to the tongue). These statistically significant differences between intervention and control were also observed in the follow-up surveys. However, after controlling for the differences already present at baseline, there was no indication that awareness of these symptoms increased at a greater rate in the intervention areas. Therefore, there was no indication that the campaign resulted in higher mention of these symptoms. Awareness of ulcers and spots was equivalent at baseline. Ulcers had higher mention among intervention respondents at both follow-ups (53 *vs* 34% (*P*<0.001) and 41 *vs* 30% (*P*<0.001), respectively); and spots had higher mention at first follow-up (14 *vs* 6% (*P*<0.001)), suggesting that these mentions had been influenced by the campaign. Awareness of lumps was lower among intervention respondents at baseline (*P*<0.05). At the first follow-up, mention of lumps did not differ significantly between intervention and control respondents, having increased among intervention respondents and having remained constant among control respondents. This increase was not maintained at the second follow-up, suggesting that the campaign had only a short-term influence on mention of lumps.

An analysis of these data by awareness of the campaign reveals that those who were aware of the campaign also have greater confidence and better knowledge of the symptoms targeted by the campaign than those who were unaware of the campaign (see Table 5). With three of the symptoms highlighted by the campaign, differences were observed between those aware and those who were unaware of the campaign at both first and second follow-up: persistent ulcers (55 *vs* 32% (*P*<0.001) and 42 *vs* 31% (*P*<0.001)), changes that persist (24 *vs* 8% (*P*<0.001) and 12 *vs* 4% (*P*<0.001)) and changes to the tongue (15 *vs* 7% (*P*<0.01) and 12 *vs* 5% (*P*<0.001)). Three other symptoms highlighted by the campaign showed a difference, by campaign awareness, at first follow-up only: spots (15 *vs* 5% (*P*<0.001)), lump(s) (14 *vs* 8% (*P*<0.05)) and a persistent red or white patch (6 *vs* 2% (*P*<0.01)). Of the two symptoms not highlighted by the campaign, bleeding and pain, the latter showed a difference at first follow-up, with those aware of the campaign less likely to identify pain as a symptom than those unaware of the campaign (12 *vs* 18% (*P*<0.01)).

### Salience of symptoms

Measures of salience were taken for two symptoms of the disease targeted by the campaign, a persistent red or white patch in the mouth and a persistent ulcer or sore in the mouth (see Table 6). At baseline, concern about each of the symptoms was the same for intervention and control respondents. At the first follow-up, concern about persistent ulcers or sores was higher among intervention respondents (77 *vs* 72% (*P*<0.05)) and this was also the case at the second follow-up (82 *vs* 71% (*P*<0.001)). Concern about persistent red or white patches in the mouth remained equal at the first follow-up, but, at the second follow-up, intervention respondents were more likely to indicate concern (82 *vs* 69% (*P*<0.001)). In both instances concern was also significantly higher among those aware of the TV advertisement at first and second follow-up.

### Behavioural intentions

A measure of intention to act was also taken for the same two symptoms. Measures of *actual* behaviour were not viable as the proportion that experienced either symptom over the study period was extremely small (1–2%). At baseline and the first follow-up, the likelihood of consulting a general medical practitioner (GMP) did not differ by intervention status. At the second follow-up, differences in intention to act between intervention and control were observed for both symptoms, with those in the intervention area significantly more likely to seek advice from a GMP than those in the control area (see Table 7). In the case of a persistent ulcer and sore, 86% of intervention respondents, compared with 77% of control respondents (*P*<0.001) said that they would consult a GMP. The equivalent figures for a persistent red or white patch in the mouth were 89 *vs* 77% (*P*<0.001).

While most (over 70%) respondents perceived it likely that they would consult a general practitioner, fewer than half (ranging from 29% to 45%) considered it likely that they would consult a dentist (see Table 7). At baseline, intervention respondents indicated being more likely than control respondents to consult a dentist regarding either of these symptoms. By the first follow-up, both groups were equally likely to consult a dentist, with likelihood having decreased among intervention respondents as baseline. At the second follow-up, likelihood did not differ from that at baseline for either group. Therefore, there is no indication of the campaign having impacted on perceived likelihood of consulting a dentist about symptoms.

An analysis of these data was also carried out by campaign awareness (see Table 7). The findings reveal that those who were aware of the campaign were more likely to respond to the symptoms than those who were unaware of the campaign: a persistent red or white patch 87 *vs* 81% (*P*<0.05) at second follow-up, and a persistent ulcer or sore 83 *vs* 77% (*P*<0.05) at first follow-up and 87 *vs* 79% (*P*<0.01) at second follow-up.

## Discussion

The results show that, even after controlling for differences at baseline and any potential influence of age, gender, social grade, marital status, education level and experience of cancer, post-campaign respondents in the intervention areas were more likely than control respondents to recall media coverage about mouth cancer, be aware of mouth cancer, recall specific symptoms featured in the campaign, be concerned about specific symptoms and indicate that they would consult a GMP about these symptoms. There was some contamination of the control respondents with 23% and 25% of the sample in the first and second follow-up, respectively, indicating awareness of the TV advertisement. As a result, it is possible that the evaluation underestimates the apparent impact of the campaign, given that some of the control sample may also be displaying signs of being influenced by the messages received. However, analysis by awareness of the TV advertisement showed that awareness of the advertisement was associated with higher awareness of symptoms of mouth cancer, increased concern about experiencing the symptoms and increased likelihood of consulting a GMP about such symptoms. Overall, the results suggest that the campaign has been successful in improving awareness of symptoms and encouraging concern and consideration of consulting a GMP should these symptoms be experienced.

Health care planners and clinicians are understandably wary of relying on mass media approaches to encourage patients to present to their GMP for advice about suspicious symptoms, fearing it could encourage inappropriate referrals and over-stretch diagnostic services. However, new learning from the development of the mouth cancer early detection campaign under investigation suggests that applying social marketing principles and involving patient groups in the development of campaign materials can ensure the message strategy used takes full account of the sensitivities surrounding the issue. The results to emerge from this evaluation provide consistent evidence for the effectiveness of using mass media to raise awareness of the symptoms of the cancer and to encourage those at risk to self-refer to a health professional for advice and diagnosis in which appropriate.

Although this study shows the effectiveness of using the mass media to promote awareness of early detection for those cancers with pre-malignant symptoms, further work is need to compare the cost-effectiveness of the self-referral model with alternative communication approaches and more conventional mass screening approaches. In particular, there is a need to examine the issue of campaign sustainability and methods for maintaining public awareness, whereas avoiding campaign and message wear out – an issue identified by other researchers looking at similar behaviour change programmes (Smith *et al*, 2002).

## Conflict of interest

The authors declare no conflict of interest.

## Figures and Tables

**Table 1:** Sample characteristics by gender, age and socio-economic status

	**Baseline**		**First follow-up**		**Second follow-up**	
	**Achieved *N* (%)**		**Achieved *N* (%)**		**Achieved *N* (%)**	
	**Intervention**	**Control**	**Intervention**	**Control**	**Intervention**	**Control**
*Gender*						
Male	255 (45)	161 (46)	273 (47)	157 (45)	273 (47)	163 (45)
Female	316 (55)	190 (54)	310 (53)	194 (55)	311 (53)	197 (55)
						
*Age*						
40–60	341 (60)	222 (63)	349 (60)	210 (60)	365 (62)	224 (62)
61–70	230 (40)	129 (37)	234 (40)	141 (40)	219 (38)	136 (38)
						
*Social grade*						
C1/C2	290 (51)	171 (49)	289 (50)	187 (53)	300 (51)	173 (48)
DE	281 (49)	180 (51)	294 (50)	164 (47)	284 (49)	187 (52)
						
Total	571	351	583	351	584	360

**Table 2:** Media coverage about mouth cancer in the last 6 months

	**Baseline**		**First follow-up**		**Second follow-up**	
**Base: all who have heard of mouth cancer**	**Intervention area (469) %**	**Control area (281) %**	**Intervention area (536) %**	**Control area (300) %**	**Intervention area (512) %**	**Control area (315) %**
Any source	27^**^	20	80^***^	26	40^***^	14
TV advertisements	2	3	74^***^	11	30^***^	3
Newspapers	2	4	7	3	5^**^	2
Radio	<1%	1	3	1	3^*^	<1%
Poster	5	4	8^**^	2	6^*^	3
Booklets/leaflets	4	2	9^**^	3	5	4

Logistic regression testing for differences by intervention status within each survey wave, controlling for gender, age, social class, marital status, education and experience of cancer. Differences are noted as follows: ^*^P<0.05, ^**^P<0.01, ^***^P<0.001.

**Table 3:** Prompted awareness of various cancers

	**Baseline**		**First follow-up**		**Second follow-up**	
**Base: all respondents**	**Intervention area (571) %**	**Control area (351) %**	**Intervention area (583) %**	**Control area (351) %**	**Intervention area (584) %**	**Control area (360) %**
Stomach cancer	90	87	90	91	91	90
Cervical cancer	91	89	91	89	89	91
Testicular cancer	87	88	88	86	89	90
Mouth/mouth cancer	82	80	92^**^	85	88	88
Ovarian cancer	84	84	85	83	86	90
Bladder cancer	61	58	65	65	69^*^	64
Bowel cancer	96	97	97	98	97	97
Breast cancer	99	98	99	99	99	99
Lung cancer	99	100	98	98	98	97
Prostate cancer	92	93	94	96	95	96
Skin cancer	97	97	97	97	97	96

Logistic regression testing for differences by intervention status within each survey wave, controlling for gender, age, social class, marital status, education and experience of cancer. Differences are noted as follows: ^*^P<0.05, ^**^P<0.01.

**Table 4:** Perceived incidence of cancer in Scotland – percent who consider each to be ‘very/quite common’

**Base: all respondents**	**Baseline**		**First follow-up**		**Second follow-up**	
**% who consider each to be very/quite common**	**Intervention area (571) %**	**Control area (351) %**	**Intervention area (583) %**	**Control area (351) %**	**Intervention area (584) %**	**Control area (360) %**
Lung cancer	90	86	88	88	88	84
Breast cancer	89	85	85	86	85	84
Prostate cancer	61	63	63	68	66	62
Bowel cancer	55^*^	61	51^**^	61	54	57
Skin cancer	59	54	49	52	46	40
Mouth cancer	23^**^	12	26^**^	15	28^**^	17
Cervical	60	57	56	55	60^*^	51
Stomach	50^**^	38	40	42	44^*^	36
Bladder	26^*^	19	21	23	30^**^	20

Logistic regression testing for differences by intervention status within each survey wave, controlling for gender, age, social class, marital status, education and experience of cancer. Differences are noted as follows: ^*^P<0.05, ^**^P<0.01.

**Table 5:** Symptom awareness

	**(a)**						**(b)**			
	**Baseline**		**First follow-up**		**Second follow-up**		**Prompted awareness of TV campaign at first follow-up**		**Prompted awareness of TV campaign at second follow-up**	
**Base: all respondents**	**Inter- vention area (571) %**	**Control area (351) %**	**Inter- vention area (583) %**	**Control area (351) %**	**Inter- vention area (584) %**	**Control area (360) %**	**Yes (563) %**	**No (371) %**	**Yes (492) %**	**No (452) %**
*Symptoms highlighted by campaign*										
Ulcers	35	31	53^***^	34	41^***^	30	55^***^	32	42^***^	31
Sores	11^*^	7	16^*^	11	15^***^	6	16	11	14^**^	9
Red or white patch	5^**^	1	6^***^	1	1	<1%	6^**^	2	1	<1%
Spots	4	3	14^***^	6	5	4	15^***^	5	5	4
Lump(s)	5^*^	9	13	9	8	6	14^*^	8	9	5
Changes that persisted (any)	6^*^	3	22^***^	11	11^***^	4	24^***^	8	12^***^	4
Change to the tongue	11^**^	4	15^**^	7	11^***^	5	15^**^	7	12^***^	5
										
*Symptoms not highlighted by campaign*										
Bleeding	17^**^	9	7^*^	4	8	6	6	6	8	6
Pain	19	17	12^*^	19	13	14	12^**^	18	15	11

Logistic regression testing for differences by (a) intervention status and (b) TV advertisement awareness within each survey wave, controlling for gender, age, social class, marital status, education and experience of cancer. Differences are noted as follows: ^*^P<0.05, ^**^P<0.01, ^***^P<0.001.

**Table 6:** Salience of specific symptoms – percent who would be ‘very/quite concerned’ if experienced the following symptoms

	**(a)**						**(b)**			
	**Baseline**		**First follow-up**		**Second follow-up**		**Prompted awareness of TV campaign at first follow-up**		**Prompted awareness of TV campaign at second follow-up**	
**Base: all respondents**	**Inter- vention area (571) %**	**Control area (351) %**	**Inter- vention area (583) %**	**Control area (351) %**	**Inter- vention area (584) %**	**Control area (360) %**	**Yes (563) %**	**No (371) %**	**Yes (492) %**	**No (452) %**
Persistent red or white patch in the mouth	66	70	80	77	82^***^	69	82^**^	74	82^***^	72
Persistent ulcer or sore in the mouth	70	65	77^*^	72	82^***^	71	79^*^	69	81^**^	73

Logistic regression testing for differences by (a) intervention status and (b) TV advertisement awareness within each survey wave, controlling for gender, age, social class, marital status, education and experience of cancer. Differences are noted as follows: ^*^P<0.05, ^**^P<0.01, ^***^P<0.001.

**Table 7:** Percent who would be ‘very/moderately likely’ to consult a GMP if experienced following symptoms

	**(a)**						**(b)**			
**Base: all respondents**	**Baseline**		**First follow-up**		**Second follow-up**		**Prompted awareness of TV campaign at first follow-up**		**Prompted awareness of TV campaign at second follow-up**	
**Very/moderately likely to consult …**	**Inter- vention area (571) %**	**Control area (351) %**	**Inter- vention area (583) %**	**Control area (351) %**	**Inter- vention area (584) %**	**Control area (360) %**	**Yes (563) %**	**No (371) %**	**Yes (492) %**	**No (452) %**
…*GMP*										
Persistent red or white patch in the mouth	80	79	82	86	89^***^	77	85	81	87^*^	81
Persistent ulcer or sore in the mouth	72	74	82	78	86^***^	77	83^*^	77	87^**^	79
										
…*Dentist*										
Persistent red or white patch in the mouth	42^*^	33	36	40	45^***^	32	39	36	45^**^	35
Persistent ulcer or sore in the mouth	42^**^	31	31	35	43^***^	29	33	31	42^**^	32

Logistic regression testing for differences by (a) intervention status and (b) TV advertisement awareness within each survey wave, controlling for gender, age, social class, marital status, education and experience of cancer. Differences are noted as follows: ^*^P<0.05, ^**^P<0.01, ^***^P<0.001.
